# Primary tumour resection plus systemic therapy versus systemic therapy alone in metastatic breast cancer (JCOG1017, PRIM-BC): a randomised clinical trial

**DOI:** 10.1038/s41416-025-03097-z

**Published:** 2025-07-04

**Authors:** Tadahiko Shien, Fumikata Hara, Kenjiro Aogi, Yasuhiro Yanagida, Michiko Tsuneizumi, Naohito Yamamoto, Hiroshi Matsumoto, Akihiko Suto, Kenichi Watanabe, Michiko Harao, Chizuko Kanbayashi, Mitsuya Itoh, Takayuki Kadoya, Keisei Anan, Shigeto Maeda, Keita Sasaki, Gakuto Ogawa, Shigehira Saji, Haruhiko Fukuda, Hiroji Iwata, On behalf of JCOG Breast Cancer Study Group, Tadahiko Shien, Fumikata Hara, Kenjiro Aogi, Yasuhiro Yanagida, Michiko Tsuneizumi, Naohito Yamamoto, Hiroshi Matsumoto, Akihiko Suto, Kenichi Watanabe, Michiko Harao, Chizuko Kanbayashi, Mitsuya Itoh, Takayuki Kadoya, Keisei Anan, Shigeto Maeda, Shigehira Saji, Hiroji Iwata

**Affiliations:** 1 Okayama University Hospital, Okayama, Japan; 2 Cancer Institute Hospital, Tokyo, Japan; 3 National Hospital Organization Shikoku Cancer Center, Matsuyama, Japan; 4 Shizuoka General Hospital, Shizuoka, Japan; 5 Gunma Prefectural Cancer Center, Gunma, Japan; 6 Chiba Prefectural Cancer Center, Chiba, Japan; 7 Saitama Prefectural Cancer Center, Saitama, Japan; 8 National Cancer Center Hospital, Tokyo, Japan; 9 Hokkaido Cancer Center, Sapporo, Japan; 10 Jichi Medical University Hospital, Tochigi, Japan; 11 Niigata Prefectural Cancer Center, Niigata, Japan; 12 Hiroshima City Hiroshima Citizen’s Hospital, Hiroshima, Japan; 13 Hiroshima University Hospital, Hiroshima, Japan; 14 Kitakyushu Municipal Medical Center, Fukuoka, Japan; 15 Nagasaki Municipal Medical Center, Nagasaki, Japan; 16 Fukushima Medical University, Fukushima, Japan; 17 Aichi Cancer Center Hospital, Nagoya, Japan

**Keywords:** Surgical oncology, Breast cancer

## Abstract

**Background:**

Several prospective studies have evaluated the benefit of primary tumour resection (PTR) in de novo Stage IV breast cancer (BC) patients, but it remains controversial. We aimed to investigate whether PTR improves the survival of de novo stage IV BC patients.

**Methods:**

De novo stage IV BC patients were enrolled in the first registration and received systemic therapies according to clinical subtypes. Patients without progression after primary systemic therapy for 3 months were randomly assigned 1:1 to systemic therapy alone (arm A) or PTR plus systemic therapy (arm B). The primary endpoint was overall survival (OS), and the secondary endpoints included local relapse-free survival (LRFS).

**Results:**

Five hundred seventy patients were enrolled between May 5, 2011, and May 31, 2018. Of these, 407 were randomised to arm A (*N* = 205) or arm B (*N* = 202). The median follow-up time of all randomised patients was 60 months. The difference in OS was not statistically significant (HR 0.86 90% CI 0.69–1.07, one-sided *p* = 0.13). Median OS was 69 months (arm A) and 75 months (arm B). In the subgroup analysis, PTR was associated with improved OS in pre-menopausal patients, or those with single-organ metastasis. LRFS in arm B was significantly longer than that in arm A (median LRFS 20 vs. 63 months: HR 0.42, 95% CI 0.33–0.53, *p* < 0.0001). There were no treatment-related deaths.

**Conclusions:**

PTR did not prolong OS. However, it improved local control and might benefit a subset of patients, such as those with premenopausal status or with single-organ metastasis. It also improved local relapse-free survival (LRFS), which is a clinically meaningful outcome in trials of systemic therapy.

**Clinical trial registration:**

UMIN Clinical Trials Registry (UMIN000005586); Japan Registry of Clinical Trials (jRCTs031180151).

## Background

De novo stage IV breast cancer accounts for approximately 6% of all newly diagnosed breast cancer cases [1]. Standard treatment includes chemotherapy, hormonal therapy, and/or radiation therapy. Primary tumour resection (PTR) is not considered a curative treatment but is sometimes performed to prevent uncontrolled chest wall disease. Some retrospective studies have suggested that PTR may improve survival in patients with de novo stage IV breast cancer [2, 3]. However, these studies have limitations, such as patient selection bias, differences in the timing of surgery, and variations in systemic therapy. Several prospective studies have investigated this question, but their results remain controversial [4–7]. It is still unclear whether PTR improves survival for any patients or whether there might be a specific subgroup that could benefit. Furthermore, there is ongoing debate about the best timing for PTR and the most effective use of systemic therapy before and after surgery. On the other hand, for patients with only a few metastatic lesions (oligometastasis), there is growing interest in an aggressive treatment approach that includes both PTR and local treatment for metastatic sites. This strategy is being actively studied in clinical trials worldwide, as it may lead to improved survival outcomes.

This trial was conducted to assess the impact of PTR after initial systemic therapy on overall survival compared to systemic therapy alone in patients with de novo stage IV breast cancer. Because patients with cancers that do not respond well to initial systemic therapy have a high risk of tumour progression during the perioperative period, only those who responded to systemic therapy were included in the randomisation.

## Methods

### Study design and participants

JCOG1017 is a multicenter, open-label, randomised, controlled phase 3 trial at 44 institutions in Japan (Appendix 1) designed to support the superiority of PTR in addition to systemic therapy for overall survival over systemic therapy alone [8]. All patients provided written informed consent before enrolment. Full trial details have been published previously; the scheme is shown in Appendix 2.

The eligible patients were aged 20–80 years with an Eastern Cooperative Oncology Group (ECOG) performance status (PS) of 0 or 1 (PS 2 caused by the symptoms of bone metastasis is also eligible.), histologically proven invasive breast cancer and without bilateral breast cancer or extension to the contralateral breast. The patients with at least one measurable metastatic lesion other than the breast tumour and axillary lymph nodes, and without brain metastasis, were included. Other eligibility criteria were no prior chemotherapy for breast cancer or radiotherapy to the ipsilateral breast, no surgery, chemotherapy or radiotherapy for any other malignancies within the past 5 years, no prior history of invasive breast cancer, and adequate organ function. Secondary registration occurred following PST for patients without progression. In addition, patients without bleeding from the primary tumour site and whose primary tumour can be completely resected were eligible. Patients judged unresectable at secondary registration were excluded.

### Randomisation and masking

Using the Japan Clinical Oncology Group (JCOG) web entry system at the JCOG Data Center, enrolled patients for the first registration receive the PST. After PST, patient’s secondary eligibility was confirmed, and patients without progression were randomly assigned (1:1) into the systemic therapy alone arm or to the PTR plus systemic therapy arm. The randomisation was conducted by the minimisation method with a random component by balancing the arms according to ER status (positive/negative), HER2 status (positive/negative), metastatic site(s) (presence/absence of visceral metastasis), and institution. Patients and all investigators were unmasked to treatment assignment.

### Procedures

Tumour measurements were conducted using contrast-enhanced computed tomography (CT) or magnetic resonance imaging (MRI). The primary and metastatic lesions were assessed prior to initial registration and again after 3 months of primary systemic therapy to determine eligibility for randomisation. All enrolled patients for the first registration received PST. PST regimen was decided according to the ER and HER2 status and the disease situation (Appendix 3). ER-positive patients, regardless of HER2 status, with no life-threatening diseases received hormonal therapy, which was oral tamoxifen 20 mg/body daily plus subcutaneous goserelin 3.6 mg/body (day 1) every 4 weeks as one cycle for premenopausal patients and oral letrozole 2.5 mg/body daily for 4 weeks as one cycle for post-menopausal patients. CDK4/6 inhibitors were not used because they were not approved in Japan during this trial period. Patients with ER-negative and/or life-threatening diseases received the following chemotherapy; for HER2-positive patients intravenous paclitaxel 80 mg/m^2^ (days 1, 8, 15) plus weekly trastuzumab 2 mg/kg (days 1, 8, 15, 22) every 4 weeks or intravenous docetaxel 75 mg/m^2^ (day 1) plus trastuzumab 6 mg/kg (day 1), pertuzumab 420 mg/body (day 1) every 3 weeks, and for HER2-negative patients intravenous paclitaxel 80 mg/m^2^ (days 1, 8, 15) every 4 weeks. After each regimen, tumour assessment was conducted after three cycles, while the docetaxel, trastuzumab, and pertuzumab regimen underwent evaluation after four cycles. In the PTR plus systemic therapy arm, the patients underwent the complete resection of the primary breast tumour within 42 days after the second registration. Both partial mastectomy and total mastectomy were acceptable as the choice of surgical procedure, so long as complete resection was possible by preoperative radiological examinations. Prophylactic axillary lymph node dissection for patients without clinically apparent lymph node metastasis by CT and /or US examination at surgery and/or wide resection of adjacent organs was not allowed. Adjuvant radiotherapy after partial or total mastectomy was not allowed. Despite the occurrence of positive surgical margins, no additional resection or radiation therapy was performed. After the second registration, the patients continued the same systemic therapy, using the systemic therapy alone arm. All randomised patients were followed for at least 4 years. Treatment for progression after the first systemic therapy was decided by physicians based on the recommendation of the Japanese guidelines [9].

### Outcomes

The primary endpoint was overall survival, the number of days from randomisation (second registration) to death from any cause, in all randomised patients. It was censored at the last follow-up date when the patient was alive. The secondary endpoints were the proportion of patients without tumour progression at the metastatic sites at 3 months after randomisation, local relapse-free survival (LRFS), the proportion of cases with local ulceration or bleeding, primary tumour resection-free survival, adverse events of systemic therapy, operative morbidity, and serious adverse events. The definition of secondary endpoints is shown in Appendix 4. Tumour progression, which was one of the eligibilities of second registration before randomisation, was defined as a 10% increase in the diameter of primary tumour and/or in the sum of diameters of target lesions, unequivocal progression of existing non-target lesions, or the appearance of new malignant lesions. Complications were evaluated using the Common Terminology Criteria for Adverse Events, version 4.0. For a post-hoc analysis, we evaluated progression-free survival.

### Statistical analysis

We assumed the median survival time was 20 months in the systemic therapy alone arm and 26 months in the PTR plus systemic therapy arm, corresponding to HR of 0.77. With the accrual period of 5 years, the follow-up period of 4 years, one-sided alpha of 5% and power of 80%, 404 patients were required. The planned number of patients of the first and the second registrations were set at 500 and 410, respectively, based on the expectation that 20% of patients registered in the first registration would not be registered in the secondary registration and a few patients would be lost to follow up.

Efficacy analyses were performed in all randomised patients based on intention-to-treat basis. The time-to-event type endpoints were estimated by Kaplan–Meier method. The primary analysis of overall survival was the log-rank test stratified with the randomisation balancing factors excluding institutions and the other *p* values were calculated by unstratified log-rank test, which were reported as two-sided. HRs were estimated by Cox’s regression, and especially for overall survival, stratified HR was estimated. For the proportion of binary endpoints, two-sided *p* values by Fisher’s exact test were calculated and Clopper and Pearson’s method estimated confidence intervals. All subgroup analyses were prespecified. The details of the accrual periods and the interim analysis were in Appendix 5. Safety was assessed in all treated patients. Statistical analyses were performed using SAS version 9.4. This study is registered with UMIN Clinical Trials Registry (UMIN000005586) and the Japan Registry of Clinical Trials (jRCTs031180151).

## Results

### Patient characteristics

Between May 11, 2011, and May 31, 2018, 570 patients were enrolled in the initial registration phase (Table 1). Among them, 294 patients (72.2%) had ER-positive breast cancer, 121 patients (29.7%) were HER2-positive, and 37 patients (9%) had triple-negative breast cancer. Regarding systemic therapy, 372 patients (65.2%) received endocrine therapy with goserelin plus tamoxifen or letrozole, 109 patients (19.1%) received systemic therapy with anti-HER2 agents such as paclitaxel plus trastuzumab or docetaxel plus trastuzumab and pertuzumab, and 88 patients (15.4%) received paclitaxel monotherapy.

**Table 1:** Patient characteristics.

		First registration	Arm A (*n* = 205)		Arm B (*n* = 202)	
		(*n* = 570)	*n*	(%)	*n*	(%)
Age median (IQR)		57 years (48–65)	57 years (49–65)		59 years (49–66)	
T1/2/3/4		35/252/69/214	8/102/26/69	3.9/49.8/12.7/33.7	17/82/22/81	8.4/40.6/10.9/40.1
N0/1/2/3		63/243/86/178	26/92/28/59	12.7/44.9/13.7/28.8	22/83/31/66	10.9/41.1/15.3/32.7
Menopausal status	Pre	198	68	(33.2)	66	(32.7)
	Post	372	137	(66.8)	136	(67.3)
ECOG PS	0	494	181	(88.3)	179	(88.6)
	1–2	76	24	(11.7)	23	(11.4)
ER status	Positive	430	149	(72.7)	145	(71.8)
	Negative	140	56	(27.3)	57	(28.2)
HER2 status	Positive	156	62	(30.2)	59	(29.2)
	Negative	414	143	(69.8)	143	(70.8)
Triple negative	Yes		17	(8.3)	20	(9.9)
	No		188	(91.7)	182	(90.1)
Metastatic site	1 organ		123	(60.0)	123	(60.9)
	>2 organs		82	(40.0)	79	(39.1)
Liver metastasis	Yes		49	(23.9)	44	(21.8)
	No		156	(76.1)	158	(78.2)
Bone metastasis	Only bone metastasis		63	(30.7)	54	(26.7)
	Not only bone metastasis		142	(69.3)	148	(73.3)

**Fig. 1:**
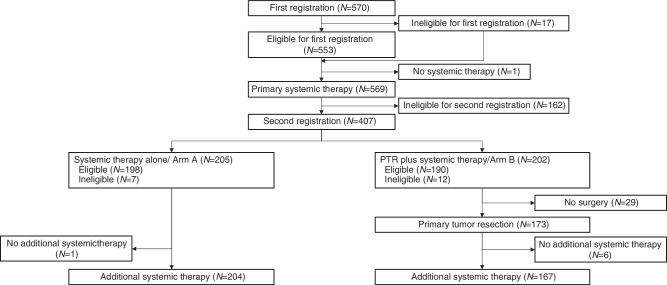
CONSORT diagram.

After primary systemic therapy, 407 patients who did not experience disease progression were randomised into two groups: the systemic therapy alone group (arm A, *n* = 205) and the PTR plus systemic therapy group (arm B, *n* = 202) (Fig. 1). The baseline characteristics were well-balanced between the two arms. Among all enrolled patients, 264 (60.4%) had metastases limited to a single organ, with the most common metastatic sites being bone (47.6%), lung (21.5%), liver (16.3%), and soft tissue (13.8%).

In arm B, 173 patients (85.6%) underwent PTR, while 29 patients (14.4%) declined surgery. During the follow-up period, 40 patients (20.2%) in arm A underwent surgery to control local disease. Details of the surgical procedures are summarised in Table 2. The median pathological tumour size was 4 cm (IQR 0–22.5), and 20 patients (11.6%) achieved a pathological complete response (pCR). Surgical margins were positive in 22 patients (13%), including 14 patients (63.6%) who underwent total mastectomy and 8 patients (36.4%) who underwent partial mastectomy. None of these patients received additional re-excision or postoperative radiotherapy to the remaining breast tissue or chest wall. Among 349 patients who terminated additional systemic therapy, the median duration after secondary registration was 10 months in arm A and 11.7 months in arm B.

**Table 2:** Operation details (*n* = 173).

	Total
	*n* = 173
Operation time	
Median (range)	90 (26–246)
Bleeding (ml)	
Median (range)	40 (0–350)
Operation (%)	
Partial mastectomy	44 (25.4)
Total mastectomy	129 (74.6)
Axillary operation (%)	
No axillary operation	75 (43.4)
Sentinel lymph node biopsy	7 (4)
Axillary lymph node dissection	91 (52.6)
Early postoperative complications	
Grade 2/3/4	11 (6.4)
Grade 3/4	2 (1.2)

No intraoperative complications were observed.

No grade 4 complications and treatment-related deaths were observed.

### Efficacy

**Fig. 2:**
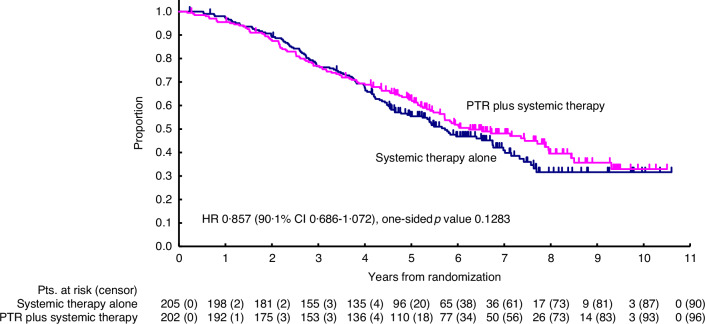
Overall survival.

The median follow-up duration for all randomised patients was 60 months (IQR 37–80). There was no significant difference in overall survival between the two arms (HR 0.86, 90% CI 0.68–1.07, one-sided *p* = 0.13) (Fig. 2). The median overall survival was 68.7 months (95% CI 55.7–81.1) in arm A and 74.9 months (95% CI 65.7–95.4) in arm B.

Seven patients in Arm A underwent early surgery, and 29 in Arm B refused surgery. A per-protocol analysis excluding these cases showed no difference in OS between groups. A related figure has been added to Appendix 6.

**Fig. 3:**
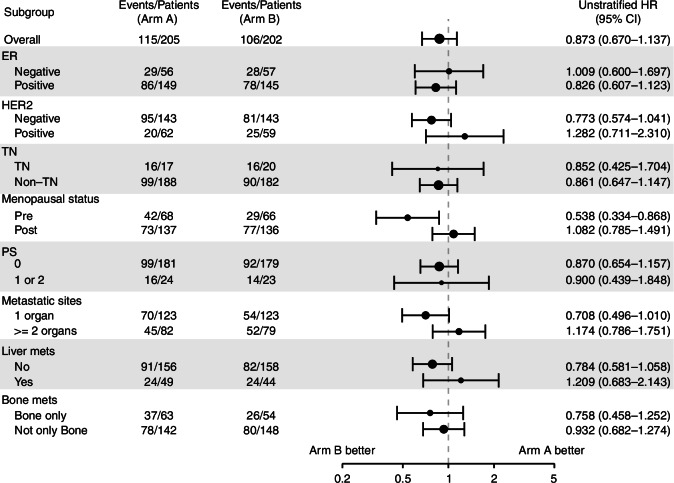
Forest plot of overall survival subgroup analyses (*n* = 407).

Planned subgroup analyses suggested that the impact of PTR might vary based on menopausal status, HER2 status, liver metastasis, and the number of metastatic organs (Fig. 3). In particular, a potential survival benefit of PTR was observed only in premenopausal patients, where the median overall survival was 76.8 months, with 65.2 months in arm A and 92.6 months in arm B. In contrast, post-menopausal patients showed no clear survival difference, with a median overall survival of 68.7 months, 71.6 months in arm A, and 68.6 months in arm B (Appendix 7).

When stratified by the number of metastatic organs, the median overall survival in patients with single-organ metastasis was 84.2 months, 76.8 months in arm A, and 95.4 months in arm B. In contrast, the median overall survival in those with multi-organ metastasis was 61.6 months, 65.1 months in arm A, and 60.1 months in arm B. However, there was no significant difference in survival based on PTR status in these subgroups. Similarly, no clear difference in survival was observed when patients were categorised by breast cancer subtype.

In arm B, patients with positive surgical margins had significantly worse outcomes than those with negative margins. The median overall survival was 60.7 months in patients with positive margins and 94.5 months in those with negative margins (HR 1.97, 95% CI 1.161–3.347) (Table 2).

**Fig. 4:**
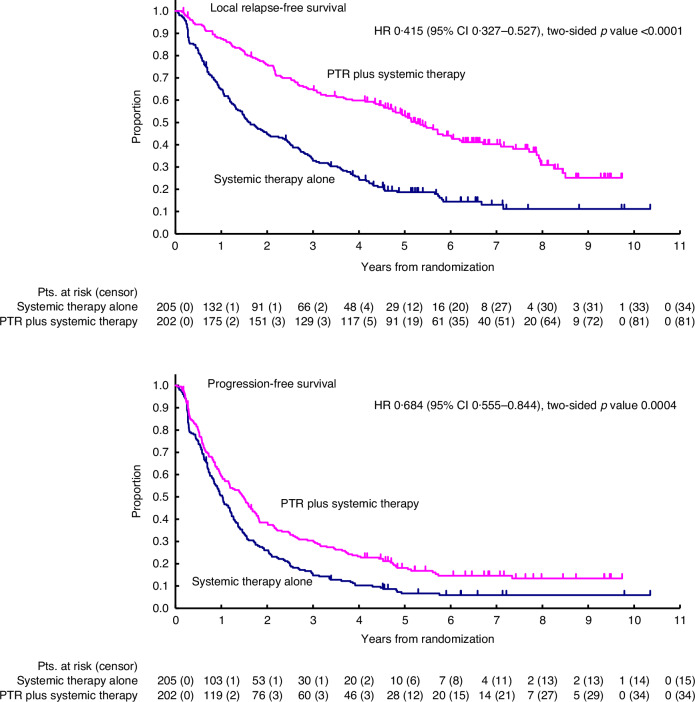
Local relapse-free survival (4-1), Progression-free survival (4-2) (*n* = 407).

Three months after randomisation, the proportion of patients without progression at metastatic sites was significantly higher in arm A than in arm B, with rates of 81.5% (95% CI 75.5–86.5) in arm A and 67.3% (95% CI 60.4–73.7) in arm B (*p* = 0.0014). Local recurrence-free survival was significantly longer in arm B than in arm A, with a median of 20 months in arm A and 63 months in arm B (HR 0.42, 95% CI 0.33–0.53) (Fig. 4). The incidence of local ulceration and bleeding was significantly lower in arm B. The 4-year cumulative incidence of local ulceration was 24.9% (95% CI 19.1–31.4) in arm A and 10.4% (95% CI 6.6–15.5) in arm B (*p* = 0.0001), while the 4-year cumulative incidence of local bleeding was 26.8% (95% CI 20.9–33.5) in arm A and 14.4% (95% CI 9.8–20.0) in arm B (*p* = 0.0021). Among the 22 patients with positive surgical margins in arm B, 10 (45.5%) experienced local recurrence after surgery.

### Safety

There was no significant difference in adverse events related to systemic therapy between the two arms. The incidence of grade 3 or higher non-haematological adverse events during systemic therapy was 6.3% (13 of 205 patients) in arm A and 5.5% (11 of 202 patients) in arm B during primary systemic therapy. During additional systemic therapy after randomisation, the incidence was 3.9% (8 of 204 patients) in arm A and 6.0% (10 of 167 patients) in arm B.

In arm B, no intraoperative complications were reported. The incidence of early postoperative complications of grade 2 or worse was 6.4% (11 of 173 patients), while the incidence of grade 3 or worse complications was 1.2% (2 of 173 patients). The grade 3 or worse complications included wound infection, wound pain, and decreased joint range of motion. No grade 4 adverse events or treatment-related deaths occurred in either group.

## Discussion

Our results show that PTR did not improve overall survival in de novo stage IV BC patients without progression to appropriate primary systemic therapy. We chose this design to tumour exclude patients not responding to primary systemic therapy in whom primary tumour resection is very unlikely to be beneficial. Even considering minimising the disadvantages of surgery, these results led us to conclude that surgery aimed at prolonging survival should not be routinely performed. This result was similar to two previously reported trials [5, 6] in which the patients who received systemic therapy were randomised. Only one trial reported the potential of the prognostic effect of surgery, but that trial had some statistical problems in randomisation and unbalanced patients’ characteristics [7]. There were more TN patients in the non-surgical arms and more patients with bone-only metastasis in the surgical arms.

When the trial was conceived, it was judged that a 6-month prolongation in OS would be clinically meaningful. The study was therefore powered, assuming a median overall survival after secondary registration of 20 months in arm A and 26 months in arm B (HR 0.77). The outcome was a 6-month difference in median overall survival in this trial (MST 69 vs. 75). However, as the median survival time of de novo stage IV BC patients in both arms was much longer than anticipated, this difference was statistically nonsignificant. A statistical reassessment showed that the sample size was sufficient to detect HR 0.77. However, proving smaller HRs to confirm the 6-month prolonging survival would have required more patients. Moreover, the study evaluated PTR after 3 months of systemic therapy, but it was not necessarily at the maximal therapeutic response. More substantial or longer initial therapy may have increased the effect of surgery. Future trials should explore optimal timing and intensity of preoperative treatment.

The Indian trial and ABCSG 28 reported worse distant progression-free survival in patients with surgery [6, 8], and a meta-analysis of prospective studies reported those results. According to Paget’s seed and soil theory [10], distant metastasis is a systemic disease. Cancer cells have already spread throughout the systemic circulation by the time distant metastases are detected. Thus, local therapies do not affect overall survival. Moreover, Fisher et al. raised the possibility that primary tumour resection may promote the progression of distant metastases [11]. This might be attributable to the resection of the primary tumour triggering surgical dissemination with increased adhesion of circulating tumour cells to the vascular endothelium of target organs, surgery-induced immunosuppression, surgery-induced angiogenic switch, or the inflammatory cascade. Additionally, effective systemic therapy during and/or after surgery might influence to progression of metastatic lesions. In Indian trials, patients did not receive effective modern systemic therapy like molecular target therapy, and the median OS was relatively shorter than in other trials. We minimised the interruption periods of systemic therapy for surgery by continuing the systemic therapy until close to surgery and restarting it within 2 weeks for endocrine therapy and 4 weeks for chemotherapy. Despite this, we observed significantly more progression at metastatic sites in the surgery arm. Additional research is needed to understand the reasons for this result and the relationship between primary tumour resection and metastatic sites.

PTR improved local control in this trial, similar to other trials [5–8]. The proportion of patients with ulcerated or bleeding primary tumours was significantly lower in the surgery arm, which may be important when making clinical decisions. There were about 25% of patients with local bleeding and/or ulceration in the non-surgery arm. Local surgery should be recommended to avoid those symptoms, but 75% do not need local surgery. Further research may allow patients at risk of these local complications to be identified. Patients who received surgery with incomplete margins had a worse prognosis than those with complete margins. A previous retrospective study reported similar results [12]. Most incomplete margins in patients with total mastectomy were on the chest wall side. In our criteria, the patients suspicious of invasion to other organs after primary systemic therapy by radiological examination were not randomised. So, these patients had muscle invasion in the primary site initially and minimal residual disease after systemic therapy, and they had a worse prognosis. In our trial, adjuvant radiation to the breast and chest wall was not allowed in order to restart systemic therapy as soon as possible. That is not clear that adjuvant radiation therapy or re-excision can avoid these worse prognoses for them. From this result, complete resection should be recommended, and further research is needed.

In preplanned subgroup analysis, PTR was associated with improved overall survival in premenopausal patients. Younger patients may be more likely to tolerate prolonged or intensified systemic treatment after surgery. MA07-01 reported similar results [7], but there was no significant difference during under 40 (HR 1.72) in E2108 [5]. We reported similar results from a retrospective study [13]. There was no evidence to confirm the difference in the meaning of breast surgery between young and elderly patients, and additional research was needed. Reducing tumour burden can improve the condition of immune function and the efficacy of drugs [14]. Volume reduction by surgery might be able to make them receive more and longer systemic therapies.

Planned subset analysis suggested that PTR may be associated with improved overall survival of patients with single organ metastasis, which is near to oligometastasis, was indicated in this study. The treatment strategy, especially local therapy, for Oligometastasis, is under debate, and there are some ongoing trials worldwide [15]. Those local therapies are included for both metastatic and breast disease if the patients were de novo stage IV BC. The prognostic effect of local therapy for patients with oligometastasis needs to be evaluated, and there are some ongoing studies to assess it. We also started registration for a new trial to confirm the prognostic effect of local therapy to both primary and metastatic sites (jRCTs031230439, NCT06135714).

Finally, the median overall survival of the de novo stage IV BC patients was prolonged by the current effective systemic therapy. There were significant differences according to the trials [5–8]. The median overall survival in the Indian trial and MF07-01 was 20 and 36 months, respectively. However, the EA 2110 and ABCSG-28 were 54 months. Moreover, the median overall survival of our JCOG1017 was 68.7 months, and the difference in overall survival between the two arms was increasing in the late period. This difference may indicate the late efficacy of surgery. Longer-time follow-up is needed to evaluate its effectiveness. Follow-up will continue with the final analysis planned 10 years after enrollment was completed in this study. Although not statistically significant, the 6-month OS difference was clinically meaningful per the original design. OS benefit was more important in favourable subgroups (e.g., single-organ metastasis, negative margins), suggesting the importance of patient selection.

### Limitations

There were limitations to this trial. The first is that systemic therapy has continued to develop, and the patients did not receive CDK 4/6 inhibitors, immune checkpoint inhibitors, and/or antibody-drug conjugates. At the time of trial initiation, immune checkpoint inhibitors and carboplatin were not approved for the treatment of metastatic breast cancer in Japan. They were not available for use in this study. Furthermore, to maintain consistency across participants and to allow accurate evaluation of the prognostic effect of primary tumour resection, chemotherapy regimens were restricted to paclitaxel monotherapy when chemotherapy was indicated. Those drugs can prolong survival for these patients, and the efficacy of PTR is unclear for them. The second is the timing of PTR. We evaluated the prognostic efficacy of PTR only after 3 months of primary systemic therapy in all subtypes. The effectiveness of systemic treatment is different according to drugs and subtypes. If the PTR should be performed at the maximum effect of drugs, most patients can get the prognostic effect, especially local control, but the timing is not unique. Moreover, this study was designed to assess the value of primary tumour resection, not complete locoregional control, including axillary dissection. To minimise delays in systemic therapy and reduce surgical complications, surgery was limited to the primary tumour, and re-excision for incomplete tumour resection was not performed. However, due to the poor prognosis observed in patients with incomplete tumour resection, the extent of nodal surgery or re-excision should be reconsidered in future studies focusing on local control.

## Conclusions

Primary tumour resection to improve survival is not recommended for de novo stage IV breast cancer. It is acceptable to perform for the purpose of local control.

## Supplementary information

Supplemental

## Data Availability

Individual participant data that underlie the results reported in this article will not be shared because the follow-up of the patients is continued until May 2028. After the publication using data as of May, 2029, individual participant data that underlie the results after deidentification will be shared if investigators whose proposed use of the data has been approved by the investigators from Breast Cancer Study Group of JCOG identified for this purpose. Proposals should be directed to tshien@md.okayama-u.ac.jp. The date will be available for achieving the aims in the approved proposal.
