# Supplementary material for: Primary tumour resection plus systemic therapy versus systemic therapy alone in metastatic breast cancer (JCOG1017, PRIM-BC): a randomised clinical trial
Source: Br J Cancer. 2025 Jul 4;133(5):625–32. doi: 10.1038/s41416-025-03097-z (PMC12405516; doi:10.1038/s41416-025-03097-z)
Supplement: Supplementary file 1 — Supplemental [file 41416_2025_3097_MOESM1_ESM.docx]

**Appendix**

1. Participating institutions of JCOG

NHO Hokkaido Cancer Center, Iwate Medical University, Tohoku University Hospital, Faculty of Medicine, University of Tsukuba, Tochigi Cancer Center, Jichi Medical University, Gunma Prefectural Cancer Center, Saitama Cancer Center, National Cancer Center Hospital East, Chiba Cancer Center, National Cancer Center Hospital, Tokyo Metropolitan Cancer and Infectious Diseases Center Komagome Hospital

National Hospital Organization Tokyo Medical Center, Showa University School of Medicine

Cancer Institute Hospital, Toranomon Hospital, St. Luke’s International Hospital, Tokai University School of Medicine, St. Marianna University, Kanagawa Cancer Center, Kitasato University School of Medicine, Yokohama Rosai Hospital, Niigata Cancer Center Hospital, Shizuoka General Hospital, Shizuoka Cancer Center, Aichi Cancer Center Hospital, Nagoya City University Hospital, Nagoya Medical Center, Kinki University School of Medicine, Osaka International Cancer Institute, Osaka National Hospital, Okayama University Hospital, Kure Medical Center Chugoku Cancer Center, NHO Fukuyama Medical Center, Hiroshima University Hospital, Hiroshima City Hospital, Hiroshima City Asa Hospital, NHO Shikoku Cancer Center, National Kyushu Cancer Center, Kitakyushu Municipal Medical Center, Nagasaki Medical Center, Kumamoto University Medical School, Social medical corporation Hakuaikai Sagara Hospital.

2. Study scheme


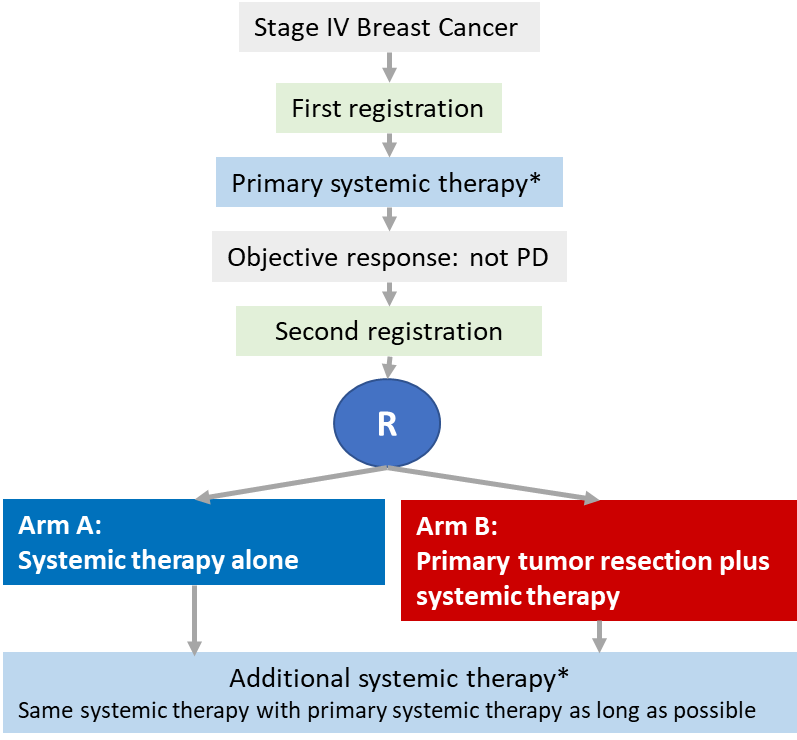


3. *Primary systemic therapy

**
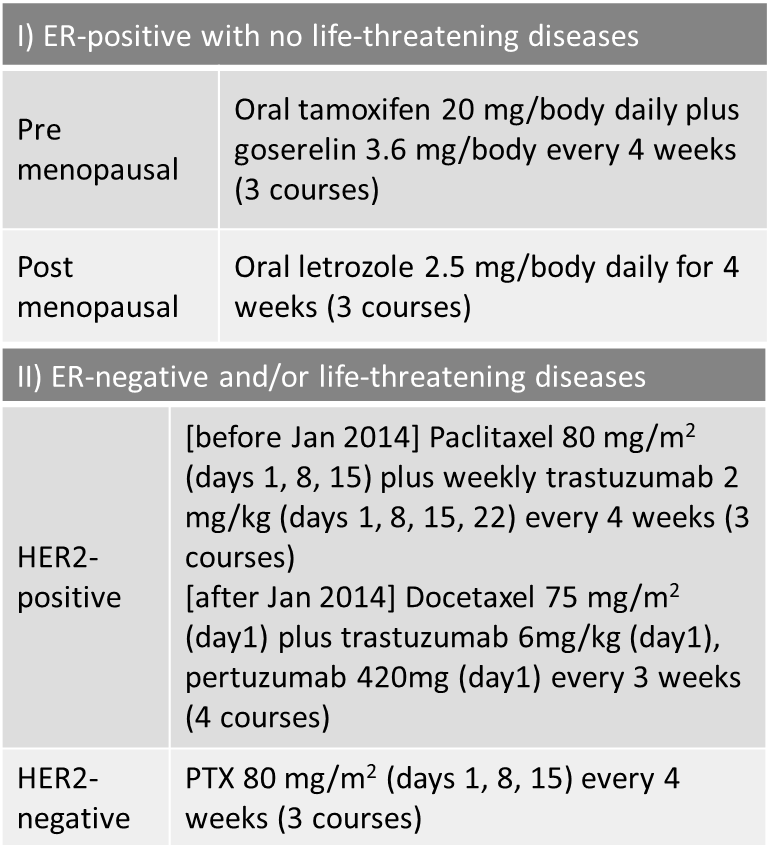
**

＊The patients with both ER and HER2-positive and non-life-threatening diseases received only hormonal therapy as primary systemic therapy; they used anti-HER2 therapy as second-line systemic therapy after progression for hormonal therapy.

** The preoperative drug interruption period was minimized. PTR was performed within 5 days of the last administration for endocrine therapy, within 14-28 days of the last administration for paclitaxel, and within 21-35 days of the last administration for docetaxel. Systemic therapy was restarted within two weeks for endocrine therapy and four weeks for chemotherapy.

**4. Definition of secondary endpoints.**

A measurable lesion is defined as a lesion that falls into one of the following categories　: (1) 20 mm or more in greatest dimension on CT or MRI in slices of 10 mm or less.　However, a CT (spiral CT) or MRI with a slice of 5 mm or less has a maximum diameter of 10 mm or more. (2) Superficial lesions (e.g., skin metastases) with a maximum diameter of 20 mm or more that can be analyzed by color photography and a measure.

LRFS was the number of days from randomization to local progression including local recurrence or death from any cause, and it is censored at the last date when the patient is alive without local recurrence. Events of PFS were in addition to the LPFS event: distant metastasis, which is the presence or absence of non-local exacerbations. Primary tumor resection-free survival was calculated in systemic therapy alone arm from the date from randomization to the date of primary tumor resection as an additional treatment or death from any cause, censored as of the last date patient was documented to be alive without primary tumor resection.

Complications were evaluated using the Common Terminology Criteria for Adverse Events, version 4.0. We used CTCAE ver. 4.0 to assess local control of tumour bleeding and tumour-induced ulceration, too.

**5. Accrual periods and interim analysis**

At Jan 13, 2015, we revised the planned number of patients of the second registration to 600, because there were more patients registered in the first registration who were not registered in the second registration than expected. Additionally, at June 10, 2016, we revised the accrual period to 7 years due to poor patient accrual.

We performed two planned interim analyses. The first interim analysis was planned to be performed once half of the planned patients had been registered in the second registration, and the second interim analysis was planned to be performed when the planned 410 patients had been registered. Lan & DeMets’ alpha spending function of O’Brien Fleming type was used to adjust multiplicity of testing overall survival . Resulting of adjusting multiplicity, the significance level for the primary analysis was 0·0497. JCOG Data and Safety Monitoring Committee independently reviewed the result of interim analyses.

**6. Overall survival of per-protocol analysis.**

**
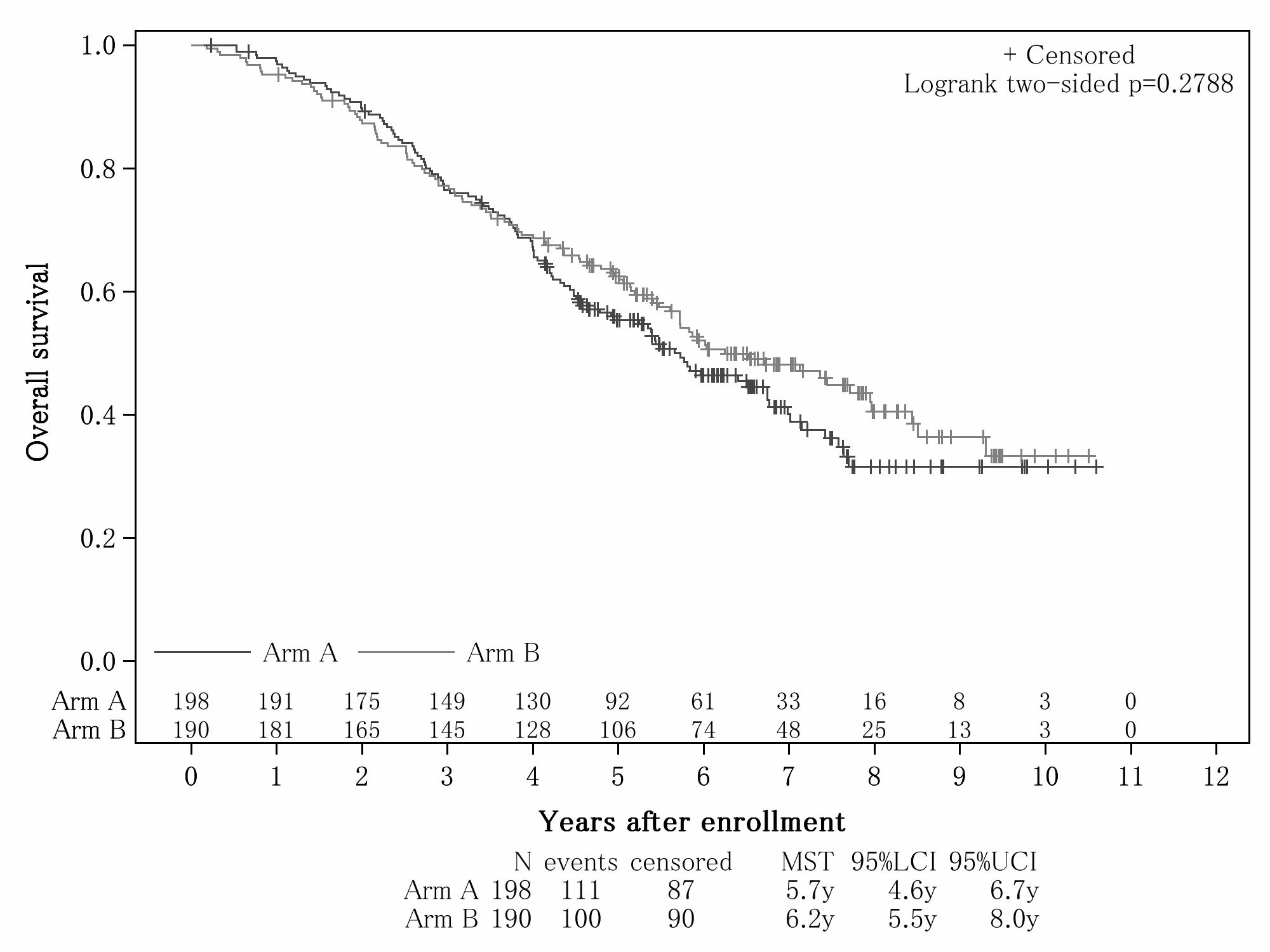
**

**7. Overall survival according to menopausal status.**

**Premenopausal patients**

**
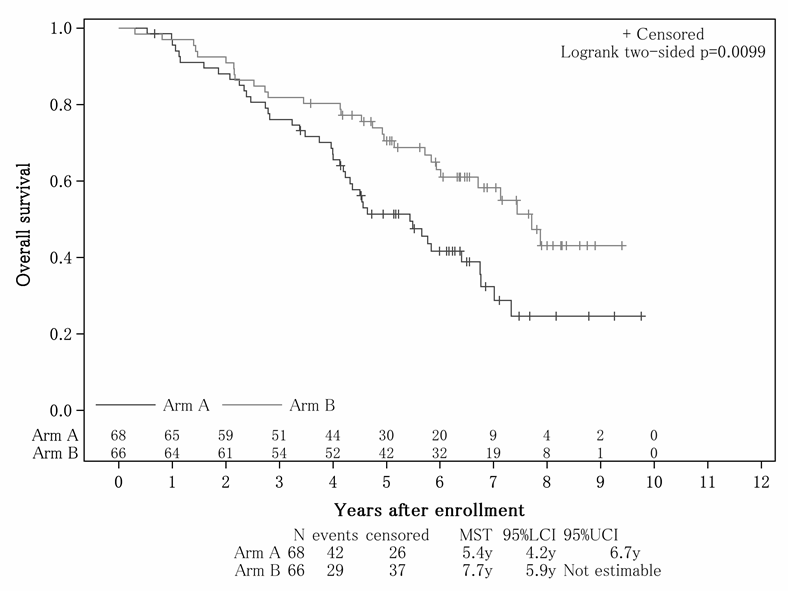
**

**Postmenopausal patients**

**
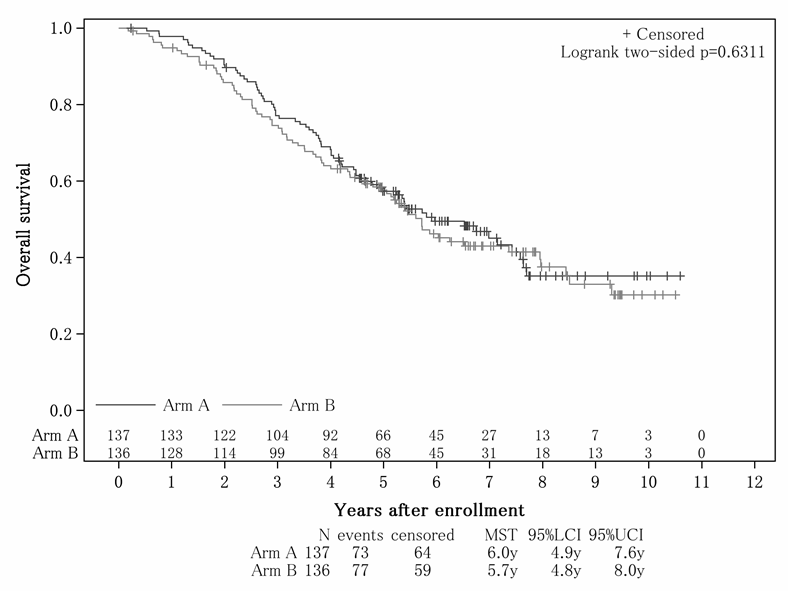
**
